# SCOFF in a general swedish adolescent population

**DOI:** 10.1186/s40337-015-0087-6

**Published:** 2015-12-15

**Authors:** Erika Hansson, Daiva Daukantaité, Per Johnsson

**Affiliations:** Kristianstad University, Kristianstad, Sweden; Lund University, Lund, Sweden

**Keywords:** Subclinical, Disordered eating behaviors, SCOFF

## Abstract

**Background:**

Although Disordered Eating Behaviors (DEB) is an ill-defined concept, multiple studies have examined prevalence of DEB and its relations to other variables in various populations. DEB have been shown to predict more serious eating disorders which in turn can lead to death. Mostly girls seem to suffer from DEB, but the question has been raised whether this, at least, partially is due to the methods used for screening. The SCOFF-questionnaire has been suggested as a quick and easily administered tool to assess DEB. However, the psychometric results regarding SCOFF suggest some inconsistencies, and more research is needed in various countries and age samples.

**Method:**

To validate SCOFF, a total of 1265 Swedish adolescents (51.6 % girls) completed self-report questionnaires using the Eating Disorder Examination Questionnaire (EDE-Q) as a reference standard.

**Results:**

The factor analyses show inconclusive results as to whether SCOFF should be regarded as a comprehensive scale; furthermore, the results indicate a correlation between SCOFF and the EDE-Q in both girl and boy samples. Girls scored significantly higher on SCOFF and also had a higher total score, indicating more severe problems than boys.

**Conclusions:**

The results raised questions as to whether the SCOFF might be interpreted and responded to in different ways by girls and boys, risking overlooking boys’ DEB and also whether one “yes” answer, instead of the stipulated two, could be sufficient when using SCOFF for screening purposes. In sum, the results challenge the use of SCOFF in a general adolescent population.

## Background

Disordered Eating Behaviors (DEB) is not a unified concept and therefore difficult to study. DEB is used to describe various behaviors or symptoms that can “take many forms” ([3], p. 41). Fasting, the use of diet pills, purging (by vomiting or the use of laxatives), and smoking for weight control are all examples of DEB [16]. DEB is furthermore suggested to have a distinct core psychopathology, with the same mechanisms in men and women, and having to do with an over evaluation of one’s own shape and weight [5].

The prevalence of DEB, despite its imprecise definition, has been calculated with frequencies varying from 15 % in boys and 33 % in girls in a study of 1895 11–17 year-old adolescents in a German sample [11] to 30 % in Israeli girls with a mean age of 14.7 years [13] to 17 % in boys and 52 % in girls with a mean age of 14.9 years in a Finnish sample [9]. The results indicate that DEB is a prevalent health problem in both girls and boys. Adolescents with DEB run a high risk of developing a clinical eating disorder [25] which renders high morbidity [12, 27] and therefore, it is of great importance to detect DEB as early as possible, providing appropriate help for adolescents at risk. For early identification of DEB, reliable and valid instruments are needed. The SCOFF-questionnaire [23] which we aim to validate in a Swedish adolescent sample in the present study, addresses core features of both anorexia nervosa and bulimia nervosa and has been used as a screening tool to enable the detection of individuals possibly at risk for an eating disorder [2, 12, 26]. During its development, great effort was made to make the SCOFF brief since it was meant to be used as part of primary care screening, often in addition to other cumbersome measures [12]. By deciding the cut-off to be the answer “yes” to two out of five questions, the researchers maximized the sensitivity of the test, which was considered a priority [12]. In the initial study by Morgan et al. [23], the test’s sensitivity in the sample of adult females already diagnosed with anorexia nervosa or bulimia nervosa was 100 %, whereas in a study by Pannocchia et al. [26], the sensitivity was 97 %. The false positive rates of 12.5 % and 12.7 %, respectively, were claimed to be an acceptable trade-off for the high sensitivity.

A validation of the SCOFF in a non-clinical adolescent sample in Finland demonstrated that 81 % of the students whose eating disorder symptoms were detected by the SCOFF-questionnaire (i.e., answered “yes” to two of five questions) had undetected DEB in a health examination performed by a nurse [10]. Furthermore, the questionnaire has been found to be accurate and reliable with a reported sensitivity of 94.6 % in a sample of students administered the SCOFF and judged to be at high or low risk of an eating disorder by a health professional [8]. Other studies are more moderate in their assessment and consider the SCOFF to have acceptable psychometric properties [15] or to be restricted but feasibly useful for ruling out the presence of eating disorders, according to a study by Lähteenmäki et al. [18] in which none of the participants with current eating disorders scored zero points on the SCOFF-questionnaire.

Thus, although multiple studies have examined the SCOFF, the results suggests some inconsistencies and more research in various samples is needed.

In addition to the SCOFF, the Eating Disorder Examination Questionnaire (EDE-Q) is a widely used instrument to examine DEB. The EDE-Q is a self-reported instrument derived from the Eating Disorder Examination interview [6] and contains questions about shape, weight and eating concerns as well as the practice of food restraint. Although the EDE-Q is a highly reliable and valid instrument in screening for DEB [20, 21], it has lately been criticized for not adequately assessing eating, weight and shape control behaviors in adolescent boys [19]. Furthermore, because of the EDE-Q’s length with thirty-six items to be answered both by multiple choice as well as with answers concerning frequencies, shorter and easier administered instruments, such as the SCOFF, which only have five items, have been requested from primary care personnel. Because the EDE-Q assesses a broad range of the specific psychopathologies of DEB, the instrument has been used as a reference in studies examining the validity of the SCOFF. It is expected that those with higher scores on the EDE-Q, indicating DEB, would also answer “yes” to two or more questions of the SCOFF.

In sum, although many of the aforementioned studies advocate the SCOFF as a useful screening tool, the results and conclusions of these studies differ. This contrast could be due to cultural influence, sample age [22], different forms of recruitment [24], or gender diversity. Because earlier adolescent validation studies have been conducted in Finland, Spain and Hong Kong, a validation study in a non-clinical, gender diversified Swedish adolescent setting would add valuable information to research regarding the validity of the SCOFF. Thus, in this study, we aim to examine the validity of the SCOFF by screening for DEB in a Swedish sample using the EDE-Q as a “reference standard”. We expect that individuals with high scores on the EDE-Q, which indicates more DEB, will also score higher on the SCOFF. Furthermore, because most validation studies contain few males and mostly adult samples, the present study will focus on adolescent boys and girls since gender differences regarding the SCOFF and its relationships to the EDE-Q also seems to be of great importance. Finally, the limit of two “yes” answers on the SCOFF is questioned, focusing on whether only one “yes” answer could be sufficient for the individual to obtain further examination to rule out DEB.

## Method

### Measures

#### Demographic variables

The demographic variables reported by the adolescents included age, gender, country of birth, country of parents’ birth, and family composition (e.g., two-parent or single-parent home).

#### Disordered eating behaviors

SCOFF. The SCOFF questionnaire [23] contains five questions concerning eating habits and attitudes toward weight and body shape: “Do you make yourself sick (vomit) because you feel uncomfortably full?”; “Do you worry that you have lost control over how much you eat?”; “Have you recently lost more than one stone (15 pounds) (ca 6.8 kg) in a 3 month period?”; “Do you believe yourself to be fat when others say you are thin?”; and “Would you say that food dominates your life?”. A threshold of two positive answers has been proposed to raise a suspicion of an existing eating disorder [17, 23].

The SCOFF was translated into Swedish by one of the authors by using back translation with help from another Swede competent in English.

EDE-Q. Twenty-two Likert-scale questions of the original 36-item version of the EDE-Q [4] were used in the present study. The questions included in the study are from the following subscales: restraint (e.g., “ How many of the past 14 days have you been deliberately trying to limit the amount of food you eat to influence your shape or weight (whether you have succeeded)”); shape concerns (e.g., “How many of the past 14 days have you had a definite desire to have a totally flat stomach”); weight concerns (e.g., “How many of the past 14 days have you had a strong desire to lose weight”) and eating concerns (e.g., “How many of the past 14 days have you had a definite fear of losing control over eating”). The questions are answered on a 7-point Likert scale ranging from 0 = “not one day” to 6 = “every day”. The questions in each subscale provide subscale scores and a global score can be calculated by summing and averaging the subscale scores. Higher scores are indicative of higher eating disorder psychopathology. In the current study, Cronbach’s alpha was .95 for the total scale, .77 for eating concern, .93 for shape concern, .85 for weight concern and .90 for restraint.

### Procedure

Both the legal guardians of students below age 15 and the students themselves, irrespective of age, received written information about the study aims, procedures and the possibility to decline participation. The students were further informed on the day of data collection and assured confidentiality. Parents provided passive consent which required them to sign and return a form if they refused to allow their child to participate in research. Students consented actively by completing the questionnaire, which took approximately 1 h. The study was approved by the Regional Ethics Committee in Lund, Sweden.

### Statistical analysis

The data for girls and boys were divided into two parts. In one of the parts, an exploratory factor analysis using principal component analysis in SPSS was conducted, and in the other part, a confirmatory factor analysis using program R was conducted to evaluate the structure of the SCOFF. The reliability of the scale was assessed with Kuder and Richardson’s formula 20 (KR20). To compare the total SCOFF-score between girls and boys, a Mann–Whitney U test was conducted, and to calculate the correlations between the SCOFF and the EDE-Q and its subscales, point biserial correlations were used. To compare the zero “yes” group of SCOFF to the one and two or more “yes” group, the non-parametric Kruskal-Wallis H test was used due to differences of variances in the groups and non-normal distribution of the scores. The pairwise comparisons were conducted by Mann–Whitney tests, and effect sizes were calculated as suggested by Field [7].

### Participants

The manuscript is based on the second wave of data collection in an ongoing longitudinal study in a municipality in southern Sweden and was conducted from January to March in 2014. The sample consisted of 1265 students (*M*_age_ = 16.19, *SD* = 1.21; 54.5 % female) who were approximately 78 % of the population of 1621 students attending the schools participating in the study. Of the 356 students who did not participate, 62 chose to refrain from participation either on their own or their parents’ initiative. The remaining 294 students were absent from school on the day of data collection for unclear reasons.

In total, 83.1 % of the adolescents were born in Sweden or another Scandinavian country, whereas the others were born in another European country (4.7 %), the Middle East (7.9 %), or other parts of the world (4.3 %). The number of foreign-born parents was somewhat higher; specifically, approximately two-thirds of the parents were born in Sweden or another Scandinavian country (mothers: 67.0 %, fathers: 67.7 %), another European country (mothers: 12.9 %, fathers: 12.4 %), the Middle East (mothers: 11.7 %, fathers: 12.0 %), or other parts of the world (mothers: 8.4 %, fathers: 8.0 %).

Approximately three-quarters of the adolescents (74.8 %) lived in two-parent households with both their biological parents, 10.3 % of the children lived in a single-parent household (8.2 % with mother), and 3.7 % lived with a parent and a step-parent (3.1 % with biological mother). One in ten adolescents (9.9 %) lived alternatingly with mother and father, 0.9 % lived with adults other than their parents, and 0.5 % lived alone.

## Results

### Exploratory factor analysis

To explore the structure of the SCOFF in the Swedish adolescent sample, a principal components factor analysis with direct oblimin rotation was conducted for half of the total sample of girls and boys separately. The Kaiser-Meyer-Olkin measure verified the sampling adequacy for the analysis of the total sample (KMO = .65) as well as for girls only (KMO = .61) and for boys only (KMO = .68). In all samples, the tests showed two factors with eigenvalues over Kaiser’s criterion of 1 explaining 56.37 % (girls: 54.61 %, boys: 60.83 %) of the variance. Table 1 shows the factor loadings after rotation for the girl and boy samples. The items that load on the same factor for the girl sample suggest that factor 1 represents thoughts about food and eating, whereas factor 2 represents actions regarding food and eating. In the boy sample, four of the items loaded on one factor and only one item; fast weight reduction, added to a second factor.

**Table 1:** Factor Loadings for Exploratory Factor Analysis with Direct Oblimin Rotation of Items of the SCOFF-scale

	Rotated factor loadings			
	Girls (*n =* 329)		Boys (*n =* 258)	
	Factor 1 Thoughts about food	Factor 2 Acts in regard to food	Factor 1	Factor 2
Do you make yourself sick (vomit) because you feel uncomfortably full?	. 23	.65	.62	.05
Do you worry that you have lost control over how much you eat?	.79	−.01	.83	−.10
Have you recently lost more than one stone (15 pounds) (ca 6.8 kg) in a 3 month period?	−.18	.84	−.01	.98
Do you believe yourself to be fat when others say you are thin?	.68	.12	.82	−.14
Would you say that food dominates your life?	.64	−.08	.53	.19

### Confirmatory factor analysis (CFA)

Based on the EFA results and to further examine the structure of the SCOFF, confirmatory factor analyses were performed to test one-factor (i.e., all SCOFF items comprise one factor) vs. two-factor models (Model 1: items 1 and 3; Model 2: items 2, 4 and 5). The results of the CFA for the second part of the participants are presented in Table 2. For girls, goodness-of-fit statistics indicated good fit for both 1-factor and 2-factor models, whereas for boys, a 1-factor model showed excellent fit with non-significant chi-square, zero value of RMSEA and high values for CFI and TLI.

**Table 2:** Summary of Confirmatory Factor Analyses for Two SCOFF Models for Girls and Boys Separately

	Girls		Boys
Goodness-of-fit statistics	1-factor model	2- factor model	1-factor model
**χ** ^2^	8.13	4.41	4.44
df	5	4	5
p	.15	.35	.49
RMSEA	.028	.018	.00
CFI	.98	.99	1.00
TLI	.97	.98	1.02
r		.61	

Based on the CFA results, the SCOFF was used as a 1-factor scale in further analyses in the present article. The reliability of the SCOFF-scale, assessed with Kuder and Richardson’s formula 20 (KR20), was .48 in the current study and considered acceptable for screening tests [28].

### Gender differences of the SCOFF

To compare the total SCOFF-score between girls and boys, a Mann–Whitney U test was conducted. The girls (*Md = .000, n = 684*) revealed higher scores, indicating higher DEB than in the boys (*Md = .000, n = 559*), *U* = 146907.5, *z* = −8.54, *p =* <.001, *r* = .24.

Table 3 shows the number and percentages of girls and boys answering “yes” and “no” to the single SCOFF-items. Large and significant gender differences indicating more “yes” answers by girls were found for all items except item 3, regarding weight loss.

**Table 3:** The number (in parenthesis) and percentages of girls and boys answering “yes” and “no” to the SCOFF items, respectively

	Total		Girls		Boys		
	“Yes”	“No”	“Yes”	“No”	“Yes”	“No”	*Z*
Do you make yourself sick (vomit) because you feel uncomfortably full?	(41)	(1238)	(33)	(645)	(8)	(545)	3.33**
	3.3 %	96.7 %	4.9 %	95.1 %	1.4 %	98.6 %	
Do you worry that you have lost control over how much you eat?	(152)	(1090)	(126)	(552)	(25)	(532)	7.52***
	12.2 %	87.8 %	18.6 %	81.4 %	4.5 %	95.5 %	
Have you recently lost more than one stone (15 pounds) (ca 6.8 kg) in a 3 month period?	(75)	(1158)	(41)	(633)	(34)	(518)	−.055
	6.1 %	93.9 %	6.1 %	93.9 %	6.2 %	93.8 %	
Do you believe yourself to be fat when others say you are thin?	(154)	(1078)	(126)	(544)	(27)	(528)	7.35 ***
	12.5 %	87.5 %	18.8 %	81.2 %	4.9 %	95.1 %	
Would you say that food dominates your life?	(180)	(1045)	(120)	(547)	(58)	(493)	3.67 ***
	14.7 %	85.3 %	18.0 %	82.0 %	10.5 %	89.5 %	

** *p* = < .001, ***p = < .0001

### Relationships between SCOFF and EDE-Q

Table 4 shows the point biserial correlations between the SCOFF and EDE-Q subscales. The results indicate strong relationships between the SCOFF and all EDE-Q subscales, with the highest relationship being eating concern for girls, *r* = .63, *p* = < .001, and weight concern for boys, *r* = .58, *p* = < .001. Although the results show significantly stronger relationships between the SCOFF and eating restraint and eating concern for girls than for boys, the differences are not large.

**Table 4:** Point biserial correlations divided between genders in regard to the SCOFF Total and EDE-Q scales

	SCOFF TOTAL		z
	Girls	Boys	
EDE-Q Total	.66 (*n* = 559)	.60 (*n* =476)	1.61 ns
EDE-Q Restraint	.55 (*n* = 619)	.39 (*n* = 521)	3.37**
EDE-Q Eating Concern	.63 (*n* = 617)	.52 (*n* = 520)	2.86*
EDE-Q Weight concern	.58 (*n* = 607)	.58 (*n* = 518)	0.00 ns
EDE-Q Shape concern	.59 (*n* = 595)	.55 (*n* = 509)	1.05 ns

Note. All reported correlations are significant at the p = .001 level (two tailed)

*p = < .01 ** *p* = < .001

### Gender differences in frequency of one “yes” answer to the SCOFF

The threshold of two “yes” answers to the SCOFF was established for further investigation of eating disorders [23]. However, one “yes”, especially on particular items (e.g., vomiting or losing a substantial amount of weight during a short period of time), may be a significant indicator of DEB risk. In the present sample, 168 of 689 girls and 85 of 575 boys answered “yes” to the SCOFF once. As Table 5 shows, one “yes” answer was rarely found on the first item regarding vomiting, indicating a non-healthy eating behavior. No significant gender differences were found on this item. More boys than girls answered “yes” on items regarding eating behavior (i.e., items three and five), whereas more girls than boys answered “yes” on items regarding losing weight and appearance.

**Table 5:** Table showing the answers posed by those answering“ yes” to only one of the SCOFF questions

	Total sample		Girls		Boys		*z*
	“Yes”	“No”	“Yes”	“No”	“Yes”	“No”	
Do you make yourself sick (vomit) because you feel uncomfortably full?	(10)	(244)	(8)	(160)	(2)	(83)	.93
	3.9 %	96.1 %	4.8 %	95.2 %	2.4 %	97.6 %	
Do you worry that you have lost control over how much you eat?	(47)	(206)	(19)	(149)	(24)	(61)	−3.39**
	18.7 %	81.4 %	11.3 %	88.7 %	28.2 %	71.8 %	
Have you recently lost more than one stone (15 pounds) (ca 6.8 kg) in a 3 month period?	(43)	(211)	(39)	(128)	(8)	(77)	2.67*
	16.9 %	83.1 %	23.4 %	76.6 %	9.4 %	90.6 %	
Do you believe yourself to be fat when others say you are thin?	(55)	(196)	(45)	(121)	(10)	(74)	2.74*
	21.9 %	78.1 %	27.1 %	72.9 %	11.9 %	88.1 %	
Would you say that food dominates your life?	(102)	(149)	(39)	(107)	(42)	(42)	−4.22*
	40.6 %	59.4 %	35.5 %	64.5 %	50 %	50 %	

*p = < .01 ** *p* = < .001*** *p* = < .0001

To further investigate differences among those with different numbers of “yes” answers to the SCOFF, girls and boys were divided into three SCOFF groups representing a group with no “yes” answers (Group 0), a group with one “yes” answer (Group 1) and a group with two or more “yes” answers (Group 2). Group differences were examined on the total EDE-Q scale and subscales, and Kruskal-Wallis’ tests were conducted to examine group differences. Pairwise comparisons were made with the Mann–Whitney test. The results are presented in Tables 6 and 7 for girls and boys, respectively. In girls, significant differences were found between all groups on all studied variables, and the effect-sizes were large when comparing group 0 to group 2. When comparing group 0 to group 1 and group 1 to group 2, the effect-sizes for the girls were in the medium range, indicating a moderate difference between the groups. The girls with one “yes” answer differed from the girls with zero “yes” answers, with effect sizes ranging from .22 for restraint to .30 for eating and weight concern. In boys, smaller effect sizes were found. However, group 1 differed significantly from both group 0 and group 2 on all EDE-Q subscales.

**Table 6:** Kruskal-Wallis Test with pairwise comparisons for the girl sample divided into three groups (Group 0 = 0 “yes”-answers to the SCOFF scale, Group 1 = 1 “yes” answer to the SCOFF-scale and Group 2 = 2 or more “yes” answers to the SCOFF scale)

Girl Sample							
	Kruskal –Wallis Test	*N*	*Mdn*	*M(SD)*	Pairwise comparisons	z***	ES
EDE-Q Total	H(2) = 174.74 ***	0 = 330	.41	.65(.68)	0–1	−6.15	.28
		1 = 137	1.09	1.32(1.10)	0–2+	−12.88	.63
		2 = 92	2.89	2.78(1.28)	1–2 +	−6.63	.44
		559					
EDE-Q Restraint	H(2) = 130.19***	0 = 361	.20	.48(.74)	0–1	−4.94	.22
		1 = 155	.40	.96(1.20)	0–2+	−11.21	.52
		2 + = 103	1.90	2.25(1.68)	1–2 +	−6.12	.38
		619					
EDE-Q Eating concern	H(2) = 188.72***	0 = 364	.00	.25(.49)	0–1	−6.73	.30
		1 = 154	.40	.76(.91)	0–2+	−13.29	.62
		2 + = 99	2.0	1.96(1.39)	1–2 +	−6.67	.42
		617					
EDE-Q Weight concern	H(2) = 170.85***	0 = 355	.40	.81(.96)	0–1	−6.66	.30
		1 = 154	1.40	1.72(1.44)	0–2+	−12.58	.59
		2 + = 98	3.20	3.11(1.45)	1–2 +	−6.14	.39
		607					
EDE-Q Shape concern	H(2) = 167.74***	0 = 352	.75	1.09(1.05)	0–1	−6.06	.27
		1 = 145	1.63	2.03(1.55)	0–2+	−12.61	.59
		2 + = 98	4.13	3.72(1.54)	1–2 +	−6.44	.41
		595					

*** *p* = < .0001

**Table 7:** Kruskal-Wallis Test with pairwise comparisons for the boy sample divided into three groups (Group 0 = 0 “yes”-answers to the SCOFF scale, Group 1 = 1 “yes” answer to the SCOFF scale and Group 2 = 2 or more “yes” answers to the SCOFF scale)

Boy Sample							
	Kruskal –Wallis Test	*N*	*Mdn*	*M (SD)*	Pairwise comparisons	*z*	ES
EDE-Q Total	H(2) = 71.87***	0 = 383	.13	.28(.44)	0–1	−5.03***	.24
		1 = 70	.45	.72(.79)	0–2+	−7.26***	.36
		2 + = 23	1.71	2.06(1.35)	1–2+	−3.77***	.39
		476					
EDE-Q Restraint	H(2) = 65.99***	0 = 419	.00	.27(.70)	0–1	−4.83***	.22
		1 = 76	.20	.76(1.25)	0–2+	−6.96***	.33
		2 + = 26	1.4	1.76(1.60)	1–2 +	−3.55**	.35
		521					
EDE-Q Eating concern	H(2) = 84.01***	0 = 420	.00	.11(.35)	0–1	−6.18***	.28
		1 = 77	.20	.37(.53)	0–2+	−7.30***	.35
		2 + = 23	1.2	1.31(1.23)	1–2 +	−3.35*	.34
		520					
EDE-Q Weight concern	H(2) = 68.03***	0 = 415	.20	.39(.65)	0–1	−3.88***	.18
		1 = 77	.60	.84(.97)	0–2+	−7.61***	.36
		2 + = 26	2.8	2.75(1.65)	1–2 +	−4.67***	.46
		518					
EDE-Q Shape concern	H(2) = 62.51***	0 = 408	.13	.43(.70)	0–1	−4.29***	.20
		1 = 74	.5	.92(.98)	0–2+	−7.03***	.34
		2 + = 27	2.25	2.59(1.85)	1–2 +	−3.80***	.38
		509					

*p = < .01 ** *p* = < .001*** *p* = < .0001

## Discussion

The aim of this study was to explore the validity of the SCOFF questionnaire [23] in a Swedish community sample of adolescents of both genders. The confirmatory factor analysis showed good fit concerning a one-factor model for girls, replicating results by Pannocchia et al. [26] and excellent fit regarding a one-factor model for boys, who were not included in the Pannocchia et al. [26] study. However, the exploratory factor analysis was not as permissive, and the results indicated that the SCOFF scale should be interpreted with some caution. Notably, the result of the exploratory factor analysis in which the scale for girls was divided into two factors (“thoughts about food” and “acts in regard to food”) resembled the two-factor model suggested by Muro-Sans et al. [24] in which the girl sample (11–17 years) loaded on the same two factors as the girl sample of the present study. Another two-factor model including girls as well as older adolescents of both genders showed a slightly better fit than a uni-factorial model [10]. The results combined indicate that although the confirmatory analysis showed good as well as excellent fit, there might be pitfalls if treating the SCOFF questionnaire as uni-factorial.

The results of the present study also showed that, consistent with earlier studies [9, 11], girls scored significantly higher on the total SCOFF and that they were over-represented in frequency in the group with 2 or more “yes” answers to the SCOFF, indicating more severe DEB. Furthermore, more girls than boys answered “yes” to the SCOFF questions concerning “lost control” and “feelings of being fat”, which implies that these questions are either indicators of more pronounced problems in girls or that the questions are interpreted differently by girls and boys. More boys than girls answered “yes” to the question whether food “dominates your life”, which may also be a question of interpretation as in either “eating all the time”, like many healthy growing adolescent boys experience that they are doing, or as constantly thinking about food and planning each calorie-intake.

Because DEB are most common among girls and the SCOFF primarily has been validated in female samples, it is possible that boys do not sufficiently adhere to the items the scale uses and that screening questionnaires in general may be missing pertinent questions for boys who might express specific “male risk behaviors” ([29] p. 448). Mond et al. [19] suggested that boys (and girls) might perform excessive exercise to lose weight. This factor could be one example of a question missing in the SCOFF. However, the question about losing a substantial amount of weight during a 3 month period might be related to this behavior. This item was also one of the questions in which girls and boys did not differ in their answers, posing the idea that this item is fairly “gender neutral”. Although hard to administer in a screening milieu, the SCOFF might benefit from a girl and boy version like the works of Kopp and Gillberg [14], investigating autism spectrum disorders in gender specific ways.

When comparing SCOFF to the EDE-Q scale, the SCOFF and the global EDE-Q were found to correlate strongly in both girls and boys. This evidence is consistent with the results reported by Leung et al. [15] using a sample of Hong Kong high school students. In evaluating the subscales of the EDE-Q, girls showed higher correlations between the SCOFF and the EDE-Q subscale restraint, which taps items concerning areas as making oneself sick. Girls also showed higher correlations than boys in regard to eating concern, which detects issues of losing control over eating and of preoccupation with food, eating and calories. Leung et al. [15] used only the global EDE-Q and therefore no further comparisons can be made; however, the results revealed in this study by the use of the EDE-Q subscales imply that when using the EDE-Q as a validation instrument, the use of the subscales would be recommended.

Although the “cut-off” for the SCOFF in an attempt to maximize both sensitivity and specificity [23] has been set at two “yes” answers, the question of whether only one “yes” might indicate DEB and warrant further investigation was assessed. As results of the present study showed, eight girls and two boys answered “yes” to the question about whether they had made themselves sick after eating too much. Although the number of adolescents who replied “yes” to the question was low, a healthy eating behavior does not involve any vomiting. Furthermore, thirty-nine girls and eight boys answered “yes” to the SCOFF question about losing 6.8 kg during a 3 month period. The answers of these two questions could be due to measuring errors but still ought to be highlighted due to the severity of the behaviors and their proximity to the DSM-V criteria of anorexia nervosa and bulimia nervosa [1], as opposed to the maybe less severe “thoughts” about food measured by the other questions. The sensitivity is undoubtedly heightened by using only one “yes” answer as cut off [8, 18, 24] but naturally, it lowers specificity. The question is whether the cost of lowered specificity is acceptable in the matter of adolescent screening.

In dividing the adolescents into three groups (zero, one and two or more “yes” answers to the SCOFF), all groups differed from each other concerning all subscales of the EDE-Q. Notably, the individuals who reported one “yes”-answer differed significantly from those reporting zero and two “yes”-answers, again indicating the importance of monitoring the “one yes” group for DEB. If the one “yes”-answer group would be investigated further this would certainly heighten the rate of false positives and perhaps lead to an attenuated questionnaire. On the other hand, since the point of SCOFF is to raise the suspicion of DEB, to hopefully get ahold of adolescent boys and girls before they develop full-blown eating disorders, this goal stands a greater chance of being fulfilled if adolescents are identified and questioned as early as possible.

### Strengths and limitations

The strengths of the present study are the large sample size and the gender diversity which provide valuable information on boys’ adherence to the SCOFF and risk of DEB as well as the differences between girls and boys in this specific area.

A limitation is that the cross-sectional design of the study only shows the scores of the SCOFF during one point in time. However, a longitudinal study using the SCOFF scale as a screening instrument might not be optimal considering the possible fluctuation of these behaviors during adolescence [10]. Still, the individuals with zero “yes” answers and those with one “yes” answer could change into two “yes” answer individuals with more severe DEB [25].

Additional limitations are the self-report format and the back translation, which was not reviewed by the original authors for face validity.

## Conclusions

Despite the above mentioned limitations, the results of the present study, in which SCOFF was highly correlated to the EDE-Q, indicate that the SCOFF is a fairly good measure of DEB. However, the exploratory and confirmatory factor analyses differed in regard to factors to be extracted and also showed different response-patterns for boys and girls.

Girls scored significantly higher on the SCOFF and also had a higher total score, indicating more severe problems than those of boys, which is consistent with the findings of earlier research. Furthermore the results raised the questions whether the SCOFF items might be interpreted differently by girls and boys and whether one “yes”-answer might be “enough” when using the SCOFF for screening purposes. Future studies should consider whether a one “yes” answer should lead to follow up questions for a more thorough screening.

Although several researchers [2, 8, 12, 26] have noted the benefits of this “simple” five-item screening-questionnaire, our results suggest that the SCOFF needs further evaluation and probably requires revision to be valid screening instrument, especially among boys. Significant gender differences on item level further indicate that more profound research is needed to reveal whether any of the questions are too blunt, vague or difficult to relate to and in which way the questions could either be reworded to fit a more gender-diversified group or entirely exchanged to fit a group of adolescent boys [19]. This information should be of interest to obtain a broader view of boys’ DEB, which might be less common but also might be undetected. Further studies are also needed to investigate whether some of the SCOFF items are critical, in which even one “yes” on the item might signal DEB risk.
